# Microbial Functional Carriers and Metabolic Background Associated with Natural Musk Quality in Forest Musk Deer

**DOI:** 10.3390/ani16213540

**Published:** 2026-11-09

**Authors:** Shuoran Sun, Zexiu Zhang, Pengcheng Yang, Sen Ye, Jingyao Hu, Jiayu Zheng, Yuetong Zhao, Xuxin Li, Runting Zhi, Xinyue Dou, Henghao Zhang, Yanru Song, Xin Dou, Xian An, Habib Bati, Shunyu Yao, Yifan Tao, Rongzeng Tan, Shuqiang Liu

**Affiliations:** 1 School of Ecology and Nature Conservation, Beijing Forestry University, Beijing 100083, China; ssr677@bjfu.edu.cn (S.S.);; 2 Fujian Pien Tze Huang Enterprise Key Laboratory of Natural Medicine Research and Development, Zhangzhou Pien Tze Huang Pharmaceutical Co., Ltd., Zhangzhou 363000, China; 3 Huang Jinming National Veteran Pharmacist Inheritance Studio, Zhangzhou 363000, China

**Keywords:** *Moschus berezovskii*, muscone, scent gland microbiota, shotgun metagenomics, untargeted metabolomics, community ecology

## Abstract

Whether differences in natural musk quality are associated with identifiable microbial ecological and metabolic configurations remains unclear, as studies integrating phenotype, community composition, functional sources, and non-muscone metabolic background are lacking. In this study, integrated metagenomic and GC–MS/LC–MS analyses were conducted on six natural musk samples from forest musk deer on a single farm, categorized into low-quality-associated (S1, S2, and S6) and high-quality-associated (S3, S4, and S5) groups. The two groups exhibited phenotypic differentiation in muscone, moisture, and sensory characteristics. Genus-level composition revealed quality-associated consortia with individual heterogeneity. KEGG Level 3 pathway decomposition into taxonomic carriers showed that similar functional potential could be achieved across genera, particularly lipid, ketone-body, and terpenoid pathways. After excluding muscone, candidate features related to lipid/membrane components, sterols, macrocyclic ketones, and volatiles changed coordinately along the quality axis. These results outline a multi-omics evidence chain of “phenotypic differentiation–consortium reorganization–functional carrier redistribution–background metabolic remodeling”, in which the identity of the organisms carrying each function provides an additional explanatory layer beyond total pathway abundance. This exploratory cross-sectional study provides testable hypotheses on the quality-associated ecological–metabolic organization of natural musk and targets for subsequent mechanistic validation.

## Simple Summary

Natural musk from forest musk deer varies in quality, but the microbial and chemical reasons for this variation are poorly understood. We asked whether the musk of different quality differs in the microorganisms living in it, in the functions these microorganisms can perform, and in its chemical make-up beyond muscone, the main fragrance compound. We studied musk from six captive male deer, three graded as high quality and three as low quality by experienced assessors at collection, by sequencing the genetic material of the whole microbial community and by two complementary chemical profiling methods. High-quality musk contained more muscone and less water, whereas low-quality musk was wetter and darker, and some samples had putrid, ammonia-like, and rancid off-odors. The two groups harbored different microbial communities; the same metabolic functions were carried by different bacteria in different animals, and many lipid, sterol, and odor-related compounds also differed. Musk quality assessment could therefore combine muscone with moisture and selected microbial and odor markers, and the musk-gland microbiota could be monitored in captive breeding; these candidate markers now need to be tested in larger groups of animals.

## 1. Introduction

Natural musk is a complex secretion produced by the musk gland sac of adult male forest musk deer (*Moschus berezovskii*). In addition to muscone, a macrocyclic ketone that imparts its characteristic aroma, natural musk contains lipids, fatty acids, sterols, and various volatile and semi-volatile components [1,2]. Musk types can vary notably in color, texture, odor, steroids, amino acids, and volatile components [3,4]. For example, normal mature musk is a brown to blackish-brown solid with a faint fragrance, whereas abnormal white musk with a sour, rotten odor and black musk with a muddy consistency and an unpleasant smell have been described in captive populations; white musk contained markedly less muscone, fewer steroid hormones, and fewer protein-derived amino acids, but more fatty acid volatiles than normal musk [3]. Across 51 musk samples from 12 farms, muscone and several steroid- and lipid-related components varied with the age, body weight, and geographic location of the deer [4]. Therefore, natural musk quality is not a linear readout of a single component but rather an integrated chemical phenotype jointly formed by the principal aroma compound, moisture status, and complex secretory matrix.

This complex phenotype is closely linked to microbial communities within the musk gland. The mating status, intraglandular maturation stage, secretion period, and musk quality are accompanied by changes in the chemical composition and community structure [5,6,7]. However, the dominant bacteria reported across studies have not been entirely consistent. Although Actinobacteria, Firmicutes, and Proteobacteria generally predominate [5,6,7], *Corynebacterium* was overrepresented in unmated males, whose musk contained more muscone [1]; *Corynebacterium*, *Proteus*, and *Atopostipes* dominated different stages of musk maturation [6]; and in a comparison of qualified and unqualified musk, *Corynebacterium* dominated both types but was more abundant in unqualified musk, whereas *Bacillus*, *Paracoccus*, and *Mycobacterium* were enriched in qualified musk [5]. In nature, various forms of symbiotic relationships exist between different members of biological systems [8,9]. The inconsistency among studies does not necessarily reduce the role of microorganisms; rather, it indicates that natural musk may be maintained by combinations of functionally complementary members, with different individuals producing similar functional outputs through different microbial configurations. Therefore, the research focus should shift from searching for a universally increasing or decreasing “core bacterium” to resolving quality-associated microbial consortia and their functional organization.

Studies on animal scent glands provide an important reference from this perspective. In systems such as spotted hyenas (*Crocuta crocuta*), striped hyenas (*Hyaena hyaena*), and giant pandas (*Ailuropoda melanoleuca*), scent gland microbial communities covary with volatile signals, and certain symbiotic bacteria generate or transform odor molecules [10,11,12,13]. These findings establish “host secretions–microbial communities–odor chemistry” as an ecological framework of broad relevance. For natural musk, the primary unresolved question is no longer limited to identifying which genera change but also which community members encode quality-associated functions and how changes in these functional sources are associated with the chemical background.

Functional redundancy theory states that the taxonomic composition of a microbial community can change substantially, whereas the overall functional repertoire remains relatively stable. The redistribution of the same function among different taxa can alter substrate competition, spatial occupancy, and metabolic exchange within a community [14]. In ruminants, integrating host phenotypes, microbial taxonomy, gene functions, and metabolites along a single evidence chain has successfully revealed the functional mechanisms underlying community composition [15]. Complex fermentation systems such as cheese and kefir also show that flavor output is frequently jointly determined by cooperation and niche partitioning among community members [16]. Together, these studies reveal a critical level of organization; identifying “who carries” the same function may more effectively explain quality differences in complex secretions than the total pathway abundance itself. Frameworks that trace community-level functional shifts back to specific taxa have shown that the taxa contributing to a function can differ markedly between functions and conditions [17]; however, such carrier-level analysis has not yet been applied to natural musk.

Based on this background, we hypothesized that (H1) natural musk of different quality differs mainly in the composition of its microbial consortia and in which genera carry key functions, rather than in overall taxonomic diversity, and that (H2) quality differences extend beyond muscone to a coordinated non-muscone metabolic background. To test these hypotheses, we first established a quality phenotype independently of the omics data, using on-site sensory grading supported by muscone and moisture measurements. We then aimed to (i) characterize community diversity and quality-associated consortia using shotgun metagenomics; (ii) decompose Kyoto Encyclopedia of Genes and Genomes (KEGG) pathways into their taxonomic carriers to determine whether the same functional potential is carried by different genera in the two groups; and (iii) characterize the non-muscone metabolic background using gas chromatography–mass spectrometry (GC–MS) and liquid chromatography–mass spectrometry (LC–MS). Finally, we integrated these layers to propose testable links among phenotype, community ecology, functional carriers, and chemical background.

## 2. Materials and Methods

### 2.1. Study Design and Sample Stratification

This study used an exploratory cross-sectional multi-omics design and included natural musk samples from six adult male forest musk deer (S1–S6; 1 sample per animal). The study was conducted in February 2026 at the Jinhengyuan Forest Musk Deer Farm in Baoding, Hebei Province, China, after the mating period of the forest musk deer. At this time of year, the musk stored in the gland sac is fully matured: in forest musk deer, musk is secreted as a liquid in early summer and gradually matures into a solid secretion by autumn [6], and February is regarded as a period when its composition is stable [3]. All samples were collected within a single harvest season (one sampling event in February 2026), because musk can be harvested from each male only at limited times of the year; as the effects on the deer of harvesting musk outside the musk-maturation period are not yet known, musk was harvested only during the traditional musk-maturation period. In addition, the study was designed as an exploratory cross-sectional comparison; between-year variation was therefore not assessed. A single farm was used so that all animals shared the same husbandry, diet, and harvesting procedure, which are known to influence musk composition [4]. All six deer were 2.5 years old and in good health, with body weights of 8.1–8.7 kg (mean ± SD, 8.43 ± 0.22 kg; Low group, 8.40 kg; High group, 8.47 kg) measured on the day of sampling. The deer were housed individually in pens of approximately 20 m^2^ under identical husbandry conditions and were fed twice daily, receiving approximately 1000 g of fresh mulberry leaves and 60 g of concentrate (corn 24 g, soybean 23 g, wheat bran 12 g, and peanut 2 g) per deer per day, with water available ad libitum; no antibiotics or related medications were used before sampling. Before sampling, the area surrounding the gland opening and the sampling tools were disinfected with alcohol. After the base of the gland had stabilized, the musk was rapidly collected using a sterile curette by a professional musk-harvesting technician. Each deer was held in a restraint frame without anesthesia, following the routine live musk-harvesting procedure of the farm; the procedure took approximately 3 min per animal, and no adverse reactions were observed during the recorded post-sampling observation. One sample of approximately 5 g was collected from each deer (six samples in total), and all samples were collected within 3 h. The samples were placed separately in sterile centrifuge tubes and immediately immersed in liquid nitrogen after the ear-tag number and sampling time were recorded. They were transported to the laboratory and stored at −80 °C; DNA extraction was completed within 1 week after sampling. Each sample was aliquoted according to a standardized procedure for moisture determination, muscone quantification, metagenomic sequencing, GC–MS, and LC–MS analyses, using the amounts described in Section 2.2, Section 2.3, Section 2.4 and Section 2.5. Sampling, liquid-nitrogen transport, and sample-processing procedures followed those of previous studies on musk from forest musk deer [3].

Quality grades were assigned on site at the time of collection, before any laboratory measurement, by ten experienced assessors (musk-harvesting technicians and quality staff of the collaborating company), who graded each sample independently according to the traditional sensory criteria used in musk production, namely color, texture, and odor. Samples that were reddish-yellow, oily in texture, and full-bodied in aroma without off-odors were graded as high quality, whereas samples that were dark and moist or had off-odors were graded as low quality; similar appearance- and odor-based criteria have been used to separate qualified from unqualified musk in previous studies [3,5]. All ten assessors independently assigned the same grade to each sample, and the written grading records were retained. Accordingly, S1, S2, and S6 were assigned to the low-quality-associated group (Low/B), and S3, S4, and S5 to the high-quality-associated group (High/Y). No numerical cut-off was predefined; muscone and moisture were measured afterwards and used to check the sensory grading quantitatively (Section 3.1). The sample order is fixed throughout the manuscript as S1, S2, S6, S3, S4, and S5. Because grouping preceded all laboratory analyses, the metabolomic and metagenomic data served to characterize and explain the grouping rather than to define it retrospectively.

### 2.2. Phenotypic Measurements and Sensory Records

Moisture content was determined by loss on drying at 105 °C. Approximately 0.50 g of fresh sample was accurately weighed and dried to a constant mass, and the moisture was calculated as the percentage of the initial mass represented by the mass difference before and after drying; three replicates were analyzed for each sample. Muscone levels were measured independently using the GC–MS external standard method. Sample preparation and instrumental conditions followed a previous procedure for analyzing musk components from forest musk deer and were optimized for the samples in this study [3]. Samples were dried to a constant mass at 60 °C, and 50 ± 2 mg was weighed, dissolved in 1 mL dichloromethane, and filtered through a 0.22 μm nylon membrane. A Shimadzu QP2010 GC–MS (Shimadzu, Kyoto, Japan) system was used with splitless injection, an injection volume of 1 μL, and a helium flow rate of 1.0 mL/min. The injector, interface, and ion-source temperatures were 250, 280, and 250 °C, respectively; the electron-impact energy was 70 eV, and the scan range was m/z 40–400. The temperature program was 80 °C for 1 min, followed by an increase to 245 °C at 5 °C/min and a 1 min hold, followed by an increase to 280 °C at 10 °C/min and a 5 min hold. An external standard curve was prepared using a muscone standard, and the peak area was converted to mass percentage. Because muscone was quantified on the dried sample, the result was converted to a fresh-mass (wet) basis using the moisture content of the same sample; wet-basis values are reported in the main text, and dry-basis values are provided in Table S4. Three replicates were analyzed for each sample. Color, texture, oiliness, and odor were recorded at collection by the ten assessors described in Section 2.1, who were unaware of any laboratory results at that time. These attributes were assessed by direct visual and olfactory inspection and recorded as categorical descriptors (color: reddish-yellow or dark; texture: oily or moist; odor: full-bodied musky aroma or off-odor); no instrumental colorimetry or olfactometry was performed. Samples S1, S2, and S6 were generally dark and relatively moist, and certain samples exhibited off-odors that the assessors described as a mixture of putrid, ammonia-like, and rancid notes, while samples S3, S4, and S5 were generally reddish-yellow, oily in texture, and full-bodied in aroma. The sensory grades defined the two groups but were not themselves subjected to statistical testing.

### 2.3. Untargeted GC–MS Analysis

The untargeted GC–MS workflow was provided by the analytical platform (Biotree Biomedical Technology Co., Ltd., Shanghai, China) and follows general procedures for GC–MS-based metabolomics [18]. A 25 ± 1 mg portion of each freeze-dried sample was weighed into a 2 mL centrifuge tube, mixed with 1.25 mL of precooled methanol/ethyl acetate extraction solution (3:7, *v*/*v*; containing 10 μg/mL n-tetracosane), and vortexed for 30 s. Following the addition of a steel bead, the sample was ground at 35 Hz for 4 min and sonicated in an ice-water bath for 5 min; this grinding–sonication cycle was repeated three times. Samples were centrifuged at 10,000 rpm (approximately 9600× *g*) for 15 min at 4 °C. A 1200 μL aliquot of supernatant was evaporated to dryness under nitrogen and reconstituted in 120 μL of n-hexane. After vortexing, samples were centrifuged again at 10,000 rpm for 1 min, and 100 μL of the supernatant was used for injection; experimental samples were analyzed in random order. This batch comprised six experimental samples. The same sample, S4, was repeatedly injected at the beginning and end of the analytical sequence (S4 and S4-2), and a blank was analyzed. Acceptance criteria were overlap in retention time and peak area in the total ion chromatograms of replicate injections, an internal-standard peak-area RSD ≤ 10%, and no significant peaks in the blank. The actual internal standard peak area relative standard deviation (RSD) was 1.84%, and the total ion chromatograms of the replicate injections overlapped well; no significant peaks were observed in the blank, and no evident systematic drift, carryover, or cross-contamination among samples was detected.

GC–MS analysis was performed using a Shimadzu GC-2030 gas chromatograph coupled to a QP2020 NX mass spectrometer (Shimadzu, Kyoto, Japan) and equipped with a DB-5MS capillary column (30 m × 250 μm × 0.25 μm; Agilent Technologies, Santa Clara, CA, USA). The injection volume was 1 μL with a split ratio of 5:1. Helium was used as the carrier gas at a column flow rate of 1 mL/min and a septum-purge flow rate of 3 mL/min. The oven temperature program was 50 °C for 1 min, followed by an increase to 310 °C at 8 °C/min and an 11.5 min hold. The injector and transfer-line temperatures were both 280 °C, and the ion source temperature was 230 °C. The electron impact energy was 70 eV; the full-scan range was m/z 50–500; the acquisition rate was 12.5 spectra/s; and the solvent delay was 2.48 min. ChromaTOF 4.3X (LECO, St. Joseph, MI, USA) was used for peak extraction, baseline correction, deconvolution, peak alignment, integration, and mass spectral matching, whereas the NIST database (2023 edition) was used for candidate identification [19].

### 2.4. Untargeted LC–MS Analysis

At low temperature, 25 ± 1 mg of freeze-dried sample was weighed into a centrifuge tube, and two homogenization beads, 500 μL of methanol/acetonitrile/water extraction solution (2:2:1, *v*/*v*/*v*), and an isotope-labeled internal-standard mixture were added. After vortexing for 30 s, the samples were homogenized at 35 Hz for 4 min and sonicated in an ice-water bath for 5 min; the homogenization–sonication cycle was repeated three times. Samples were maintained at −40 °C for 1 h and then centrifuged at 12,000 rpm (approximately 13,800× *g*) for 15 min at 4 °C; the supernatant was collected for analysis. Polar metabolites were analyzed using a Vanquish ultra-high-performance liquid chromatography system (Thermo Fisher Scientific, Waltham, MA, USA) and a Waters ACQUITY UPLC BEH Amide column (2.1 × 100 mm, 1.7 μm) (Waters Corporation, Milford, MA, USA). Mobile phase A was an aqueous solution containing 25 mM ammonium acetate and 25 mM ammonium hydroxide (pH 9.75), and mobile phase B was acetonitrile. Nonpolar metabolites were analyzed using a Kinetex C18 column (2.1 × 100 mm, 2.6 μm; Phenomenex, Torrance, CA, USA). Mobile phase A was 0.01% aqueous acetic acid, and mobile phase B was isopropanol/acetonitrile (1:1, *v*/*v*). The autosampler temperature was 4 °C, and the injection volume was 2 μL.

Mass spectrometric detection was performed using an Orbitrap Exploris 120 instrument (Thermo Fisher Scientific, Waltham, MA, USA) controlled by Xcalibur 4.4. The sheath-gas and auxiliary-gas flow rates were 50 and 15 Arb, respectively, and the capillary temperature was 320 °C. The full-scan and MS/MS resolutions were 60,000 and 15,000, respectively, and the stepped normalized collision energies were 20/30/40. The spray voltages in positive- and negative-ion modes were 3.8 and −3.4 kV, respectively. Raw data were converted to mzXML format using ProteoWizard 3.0.24054 [20], after which peak detection, extraction, retention time correction, alignment, and integration were performed using an in-house R program developed based on XCMS (version 4.2.2) [21]. Candidate metabolites were annotated using BiotreeDB 4.0 and a knowledge-guided multilayer metabolic-networking workflow [22]. Annotation levels retained the original labels in the source data table, and candidate annotations were not promoted to compounds confirmed by authentic standards.

### 2.5. Metagenomic Sequencing and Basic Processing

Total DNA was extracted using an OMEGA Soil DNA Kit (D5635-02) (Omega Bio-tek, Norcross, GA, USA), and DNA integrity was assessed using an Agilent 5400 (Agilent Technologies, Santa Clara, CA, USA). For each sample, 1 μg of genomic DNA was fragmented to approximately 300–350 bp by Covaris ultrasonication (E220 Focused ultrasonicator; Covaris, LLC, Woburn, MA, USA), followed by end repair, A-tailing, adapter ligation, purification, and PCR amplification. AMPure XP/SPRISelect was used for the purification and fragment selection. Libraries were quantified using a Qubit 3.0 (Thermo Fisher Scientific, Waltham, MA, USA); insert sizes were assessed using an Agilent 5400, and qPCR was used to confirm an effective library concentration of >3 nM. Qualified libraries underwent paired-end 150 bp sequencing on the Illumina NovaSeq X Plus platform (Illumina, San Diego, CA, USA).

Adapters were removed from raw reads using fastp 0.23.0, and reads < 50 bp with an average quality of <20 or those containing ambiguous bases were filtered out [23]. The reads were then aligned to the host reference genome using BWA 0.7.9a, and host-derived reads were removed [24]. Clean reads were assembled using MEGAHIT 1.2.9, and contigs ≥ 500 bp were retained [25]. Open reading frames were predicted using Prodigal (version 2.6.3), and genes ≥ 200 bp were retained [26]. The non-redundant gene set was clustered using CD-HIT 4.8.1 with 95% nucleotide identity and 90% coverage [27]. Reads were mapped back to the gene set using Bowtie2 (version 2.5.3) with 95% identity, and the transcripts/mapped reads per million normalized abundance (TPM) was calculated [28].

### 2.6. Taxonomic and Functional Annotation and the Functional Carrier Matrix

Representative gene protein sequences were aligned against the NR database using DIAMOND 2.0.13 (E ≤ 1 × 10^−5^) for taxonomic annotation [29]. Functional annotation included the eggNOG 4.5.1/eggNOG-mapper workflow (E ≤ 1 × 10^−5^) [30], KEGG 2023-08-30 (E ≤ 1 × 10^−5^) [31], and CAZy/hmmscan (CAZy: V8, HMMER 3.4) (E ≤ 1 × 10^−5^) [32]. The KEGG Orthology (KO), Level 3 Pathway, EC, and module abundances were calculated by summing the TPM values of the corresponding genes. The KEGG results represent the functional potential encoded by the metagenome.

The primary functional level of the functional carrier matrix was the KEGG Level 3 pathway, and the taxonomic carrier level was the genus. If a gene mapped to *n* Level 3 pathways, its TPM was divided equally by 1/n before aggregation to prevent multiple counts. For each sample and pathway, the contribution proportion of each genus was calculated as the TPM assigned from that genus to the pathway divided by the total TPM of the pathway in the sample. The mean sample contribution proportions were calculated separately for the High and Low groups, where High−Low represents a change in the carrier share. Differences in pathway carrier profiles were described using a Bray–Curtis-type distance, Σ|Highi − Lowi|/Σ(Highi + Lowi). The matrix contained 988 genera, 415 Level 3 pathways, and 18,778 genus–pathway combinations; no between-group significance tests were performed for pathway carriers.

### 2.7. Community Diversity, Composition Analysis, and Stability Assessment

Shannon diversity and Simpson dominance at the genus and KO levels were calculated from TPM proportions. Because genus composition is subject to a constant-sum constraint, a pseudocount of 1.78 × 10^−9^ was added to the 1686 detected features before centered log-ratio transformation, and principal component analysis was performed in Aitchison space [33]. The stable-feature display matrix retained 96.7–99.5% of the genus-level relative abundance in each sample and was renormalized to 100%. The unclassified sequences were separated from all other classified genera. The signs of the principal component analysis (PCA) axes indicate only the display orientation and do not alter the distance between samples.

For community and functional indices, all 3-versus-3 groupings were enumerated, and two-sided exact permutation *p* values were calculated. Because the smallest attainable two-sided *p* value at *n* = 6 was 0.10, between-group comparisons incorporated both effect direction and leave-one-out (LOO) stability. The LOO analysis recorded the number of times the High−Low direction was retained after excluding each sample to assess the sensitivity of between-group trends to individual samples. Focal genera were selected based on between-group effect magnitude, directional stability, and consortium representativeness. Carrier pairs were selected based on changes in contribution share, High/Low ratio, and stability tier. To evaluate whether the observed grouping was exceptional relative to chance partitions, the main multi-layer summary statistics (genus-level separation in Aitchison space, number of genera with completely separated group values, mean carrier-profile distance of the five core pathways, and metabolome-wide separation of the non-muscone features) were recomputed for all ten unique 3-versus-3 partitions of the six samples, and the rank of the observed grouping was recorded (Table S5). Separation was quantified as the PERMANOVA pseudo-F statistic computed from Euclidean distances. Effect sizes of the directly measured phenotypes were expressed as Hedges’ g; for a 3-versus-3 design, a two-sided *t*-test reaches 80% power at α = 0.05 only for standardized differences of approximately d ≥ 3.1.

### 2.8. Background Metabolome and Differential Features

In total, 116,658 untargeted metabolic features were detected. Before pre-processing, the muscone feature, which is a grouping phenotype, was removed. The remaining 116,657 features were transformed using log2(value + 1.18 × 10^−9^) and then Z-standardized by feature across the six samples. Confidence in metabolite annotations was classified according to the Metabolomics Standards Initiative and high-resolution mass spectrometry annotation frameworks [34,35,36]. Except for Level 1 confirmation with authentic standards, all compound names were treated as candidate annotations.

Differential features were initially screened using *p* < 0.05 and variable importance in projection (VIP) > 1, and fold changes were subsequently calculated. The Benjamini–Hochberg method was used to control for the false discovery rate (FDR) [37]. Representative candidates were selected by jointly considering the annotation confidence, chemical-class relevance, directional stability between groups, and cross-layer explanatory values.

### 2.9. Cross-Omics Integration and Statistical Analysis

Cross-layer integration focused on three independent and traceable classes of evidence: directly measured quality phenotypes, genera and their functional carrier shares, and background metabolic features after muscone exclusion. The High−Low direction was first compared across layers, after which results with consistent directions and explicit sources were summarized in a cross-sectional evidence map. Spearman’s correlations and existing network analyses were used to extend the exploratory cross-layer associations. The final R data organization and visualization components used were R 4.6.1 (aarch64-apple-darwin23; macOS Sequoia 15.3).

### 2.10. Ethical Considerations

This study was conducted in accordance with the guidelines of the Animal Care and Use Committee of Beijing Forestry University, and sampling and experimental protocols were approved by the relevant ethics committee (approval number: EAWC_BJFU_2024021). Sample collection was authorized by the farming facility; musk was collected by routine live harvesting under physical restraint without anesthesia, and no additional invasive procedures were performed on the animals during the experiment.

## 3. Results

### 3.1. Opposing Changes in Muscone and Moisture Define a Distinct Quality Phenotype

**Figure 1:**
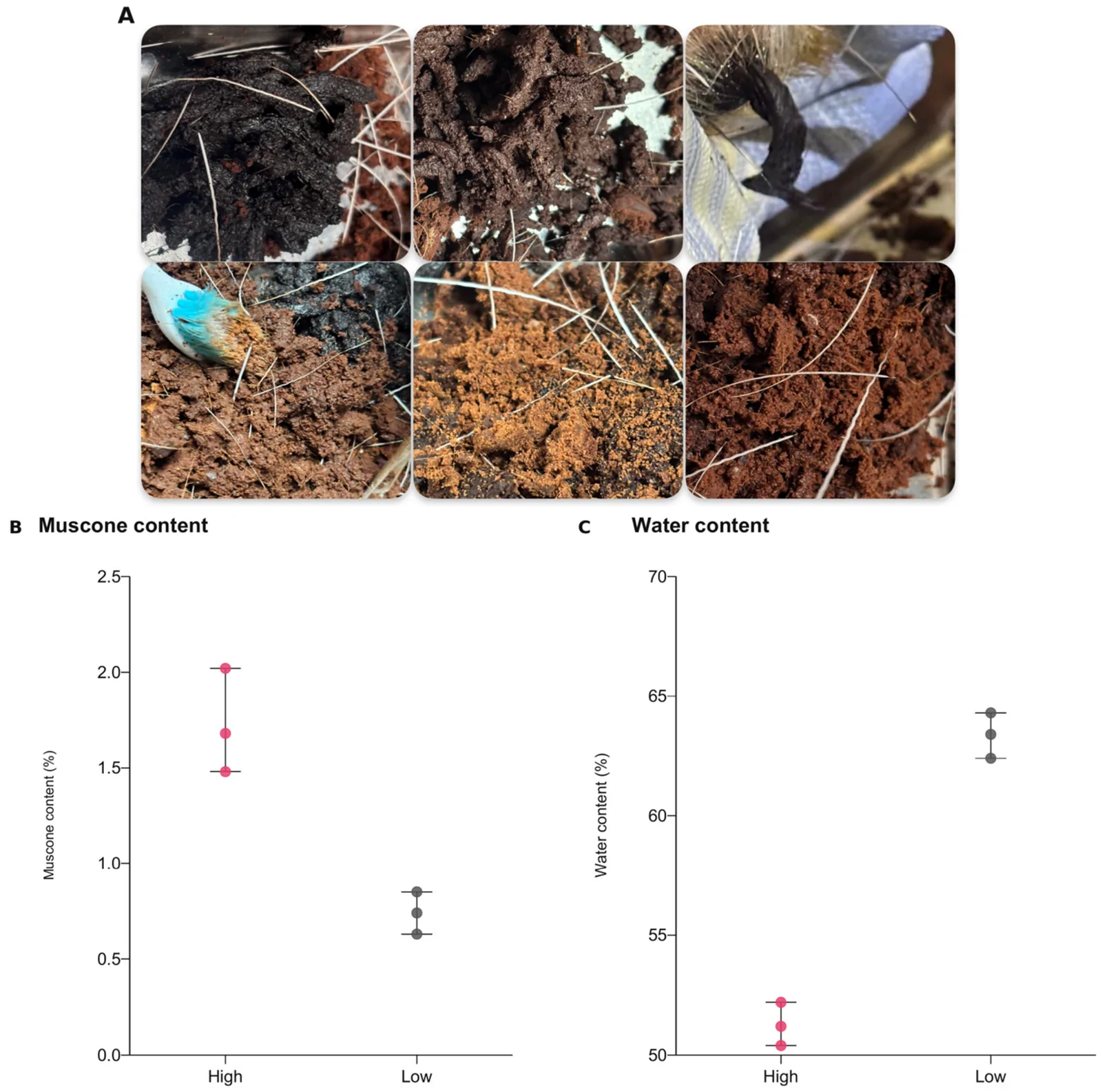
Appearance and directly measured quality phenotypes of natural musk: (**A**) photographs of samples arranged as S1, S2, S6, S3, S4, and S5; (**B**) muscone content; (**C**) moisture. The High/Y group (pink) includes S3, S4, and S5, and the Low/B group (gray) includes S1, S2, and S6. Points represent individual samples, and horizontal lines represent within-group summaries; muscone and moisture are both expressed as fresh-mass (wet-basis) percentages.

Two directly measured indices were consistent with the on-site sensory grading (Figure 1). The muscone contents of S1, S2, and S6 were 0.69%, 0.78%, and 0.86%, respectively, with a group mean of 0.78%, whereas those of S3, S4, and S5 were 1.51%, 1.80%, and 2.04%, respectively, with a group mean of 1.78%. The high-quality-associated group showed an average increase of 1.0 percentage point (Hedges’ g = 4.1) relative to the low-quality-associated group, and this trend was retained in all six leave-one-out analyses. When expressed on a dry-mass basis, muscone ranged from 1.91% to 2.40% in the Low group and from 3.06% to 4.23% in the High group, so the two groups remained completely separated (Table S4).

Moisture exhibited an opposite and equally stable change relative to that of muscone. The moisture contents of S1, S2, and S6 were 63.8%, 62.1%, and 64.2%, respectively, with a group mean of 63.4%. Those of S3, S4, and S5 were 50.7%, 52.1%, and 51.8%, respectively, with a group mean of 51.5%, representing a between-group decrease of 11.8 percentage points (Hedges’ g = −10.0). The LOO stability was 6/6. The resulting “high-muscone–low-moisture” combination was highly consistent with sample appearance and odor: high-quality-associated samples were reddish-yellow, oily in texture, and full-bodied in aroma, whereas low-quality-associated samples were typically darker and had a higher moisture content, with some exhibiting putrid or other undesirable odors. This opposing phenotype established an explicit quality coordinate for subsequent omics comparisons.

### 3.2. Taxonomic Diversity Retains Individual Differences, Whereas the Functional Gene Repertoire Expands Consistently Along the Quality Axis

Genus-level α-diversity showed notable individual heterogeneity. The Shannon indices of the Low and High groups were 2.83 and 2.95, respectively (Δ = +0.12, exact *p* = 0.80, LOO 4/6), and Simpson dominance values were 0.171 and 0.167, respectively (Δ = −0.003, *p* = 1.00, LOO 4/6). S1 had the lowest Shannon index (2.25), whereas S6, also in the Low group, had the highest (3.58). Therefore, the difference between the two types of musks did not manifest as a simple overall increase or decrease in taxonomic richness but rather in differences in community composition.

In contrast, KO-level diversity and richness changed consistently along the quality axis. The KO Shannon indices of the Low and High groups were 6.72 and 6.88, respectively (Δ = +0.16, exact *p* = 0.10), and Simpson dominance values were 0.0019 and 0.0015, respectively (Δ = −0.0004, *p* = 0.10). The number of detected KOs increased from 1911 to 2434 (Δ = +523, *p* = 0.10). The High−Low directions of all three indices were retained in all six LOO analyses, indicating that the functional gene repertoire of the high-quality-associated samples was broader and more evenly distributed. Because 0.10 is the smallest attainable two-sided exact *p* value in a 3-versus-3 design, the observed grouping was the most extreme of all possible partitions for these KO-level indices.

**Figure 2:**
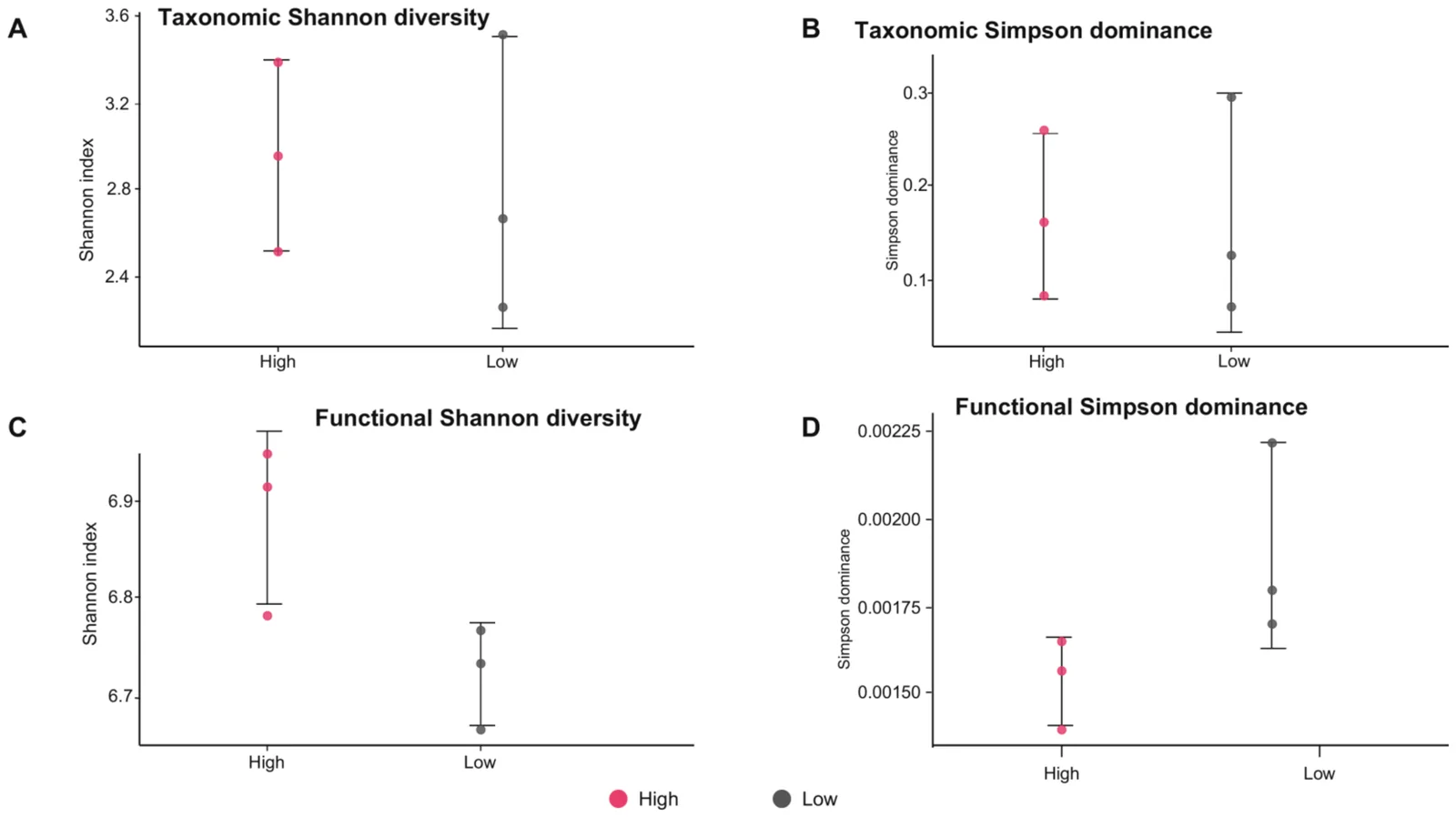

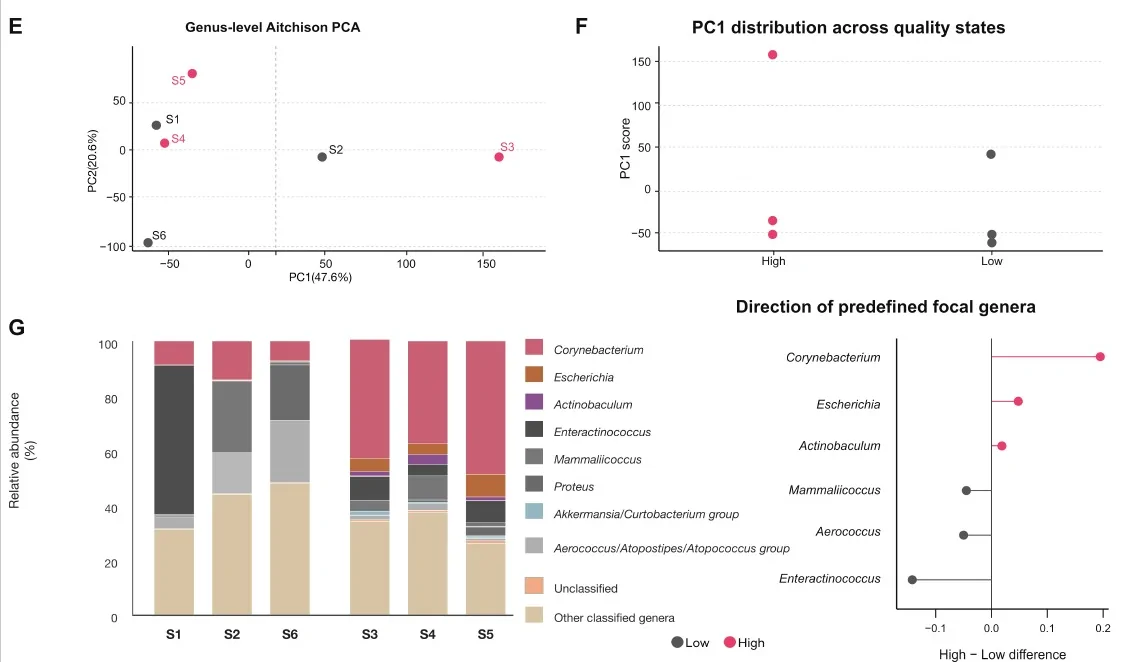
Taxonomic diversity, functional diversity, and quality-associated consortia of the natural musk microbial community: (**A**,**B**) genus-level Shannon diversity and Simpson dominance; (**C**,**D**) KEGG Orthology (KO)-level Shannon diversity and Simpson dominance; (**E**,**F**) genus-level Aitchison principal component analysis (PCA) and the between-group distribution of PC1; (**G**) composition of nine focal genera in the fixed sample order and their High−Low directions. The composition plot shows members of the quality-associated consortia, with unclassified sequences and all remaining classified genera summarized separately; points represent individual samples.

Aitchison PCA further revealed the coexistence of a quality axis and individual differences. Principal components (PC1 and PC2) explained 47.6% and 20.6% of the community variation, respectively. S3 was notably displaced toward the positive end of PC1 (159.3), whereas S4 and S5 had scores of −52.5 and −35.9, respectively. In the Low group, S2 scored 47.7, whereas S1 and S6 scored −56.5 and −62.2, respectively (Figure 2). This spatial structure indicates that quality stratification captures a major axis of community organization; however, different samples can reach similar quality states through different taxonomic combinations.

### 3.3. Quality Differentiation Corresponds to Combinatorial Reorganization of Microbial Consortia

Nine focal genera separated into two opposing sets along the quality axis (Figure 2G). *Corynebacterium*, *Escherichia*, *Acinetobacter*, and *Actinobaculum* were more abundant in the High group, whereas *Enteractinococcus*, *Proteus*, *Aerococcus*, *Mammaliicoccus*, and *Atopococcus* were more abundant in the Low group. The strongest signals were *Corynebacterium*, with a mean relative abundance of 10.0% in the Low group and 28.9% in the High group, and *Enteractinococcus*, with 18.3% and 5.2%, respectively; the complete group means and differences are provided in the source data.

These group means arose from different dominant members in different samples. *Corynebacterium* accounted for 37.6% and 48.7% of the communities of S4 and S5, respectively, but only 0.5% of S3, whereas the Low-group samples S1 and S6 were dominated by *Enteractinococcus* (54.4%) and *Proteus* (20.2%), respectively. The High−Low direction of *Corynebacterium* was retained in all six LOO analyses, but only six of the nine cross-group sample pairs supported it, indicating that its contribution was embedded in sample-specific community backgrounds.

Thus, the quality-associated taxonomic signal was not driven by a single genus that changed synchronously across all samples but by several interchangeable dominant members whose combined direction remained consistent. This pattern of interchangeable members within a consistent consortium direction motivated the subsequent analysis of which genera carry each function.

### 3.4. Pathway Functions Undergo Carrier Redistribution Between Quality-Associated Consortia

**Figure 3:**
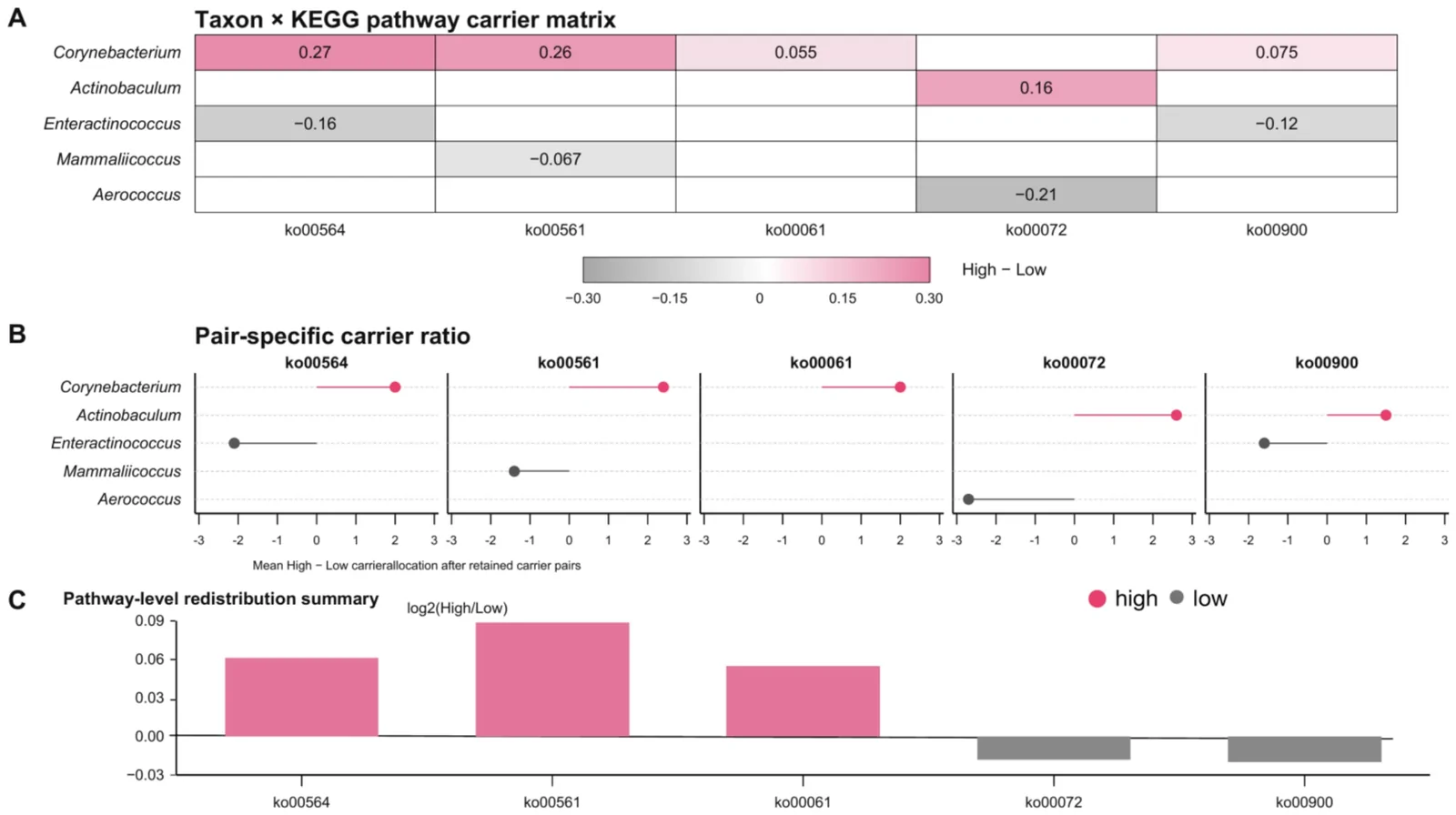

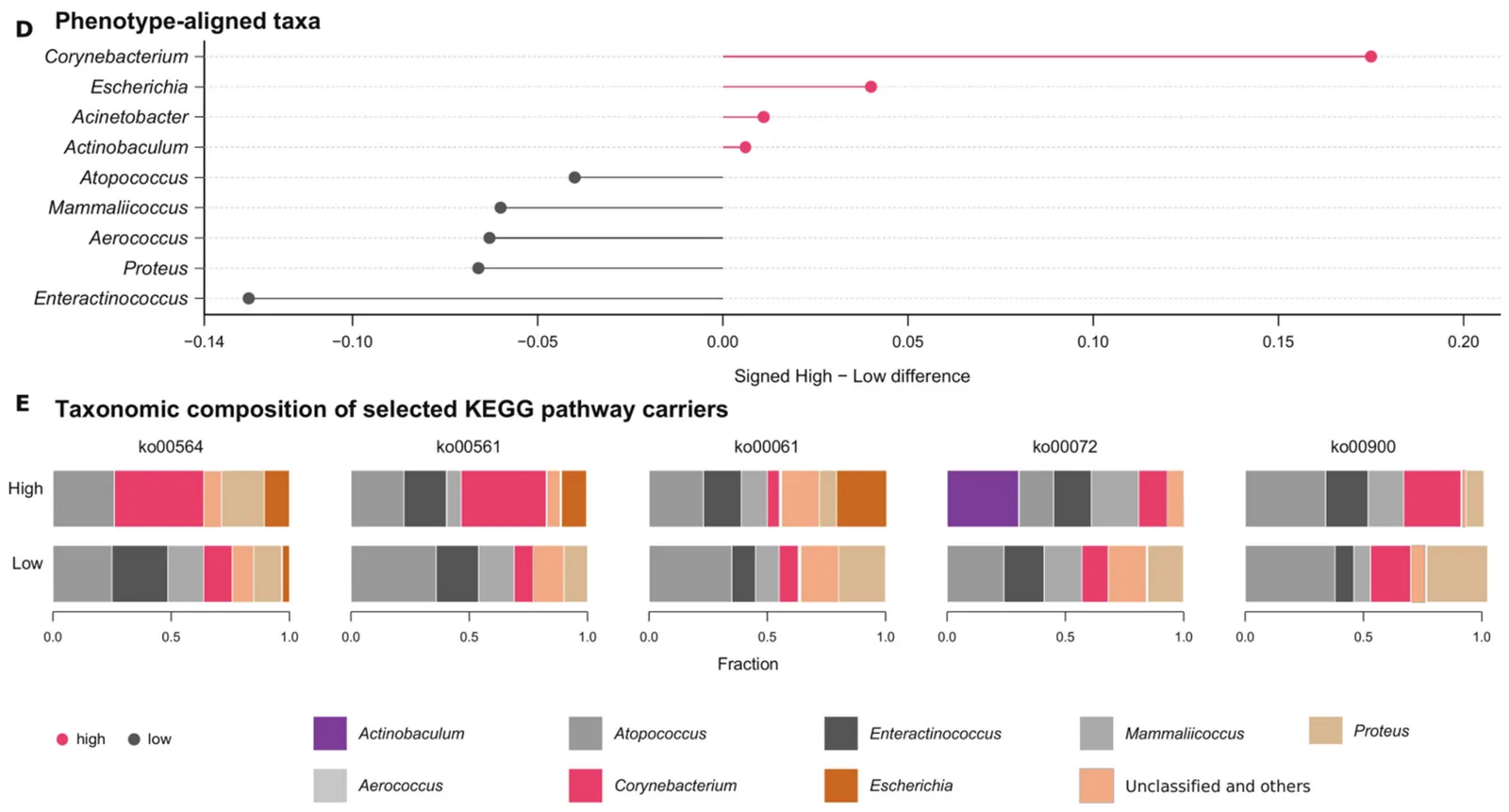
Redistribution of Kyoto Encyclopedia of Genes and Genomes (KEGG) Level 3 pathway carriers within quality-associated consortia: (**A**) genus × pathway carrier matrix, with values representing High−Low contribution shares; (**B**) log2(High/Low) carrier ratios for genus–pathway pairs; (**C**) mean redistribution magnitude at the pathway level; (**D**) taxonomic abundance directions of focal genera; (**E**) mean carrier composition of five core pathways in the High and Low groups. Carrier shares were calculated from metagenomically encoded functional potential; blank cells indicate genus–pathway combinations not included in the display.

Decomposing total pathway abundance by genus of origin showed that the genera carrying the five core KEGG Level 3 pathways differed between the two groups (Figure 3). The distances between the group-mean carrier profiles were 0.61 for glycerophospholipid metabolism (ko00564), 0.58 for glycerolipid metabolism (ko00561), 0.45 for fatty acid biosynthesis (ko00061), 0.45 for synthesis and degradation of ketone bodies (ko00072), and 0.55 for terpenoid backbone biosynthesis (ko00900).

*Corynebacterium* was the main High-quality-associated carrier. Its shares of ko00564, ko00561, ko00061, and ko00900 increased from 0.13, 0.08, 0.02, and 0.09 in the Low group to 0.41, 0.34, 0.08, and 0.16 in the High group (1.8- to 4.2-fold). For ko00072, the main High-quality-associated change was an increase in the share carried by *Actinobaculum*, from 0.04 to 0.20 (5.6-fold).

Conversely, the main Low-quality-associated carriers contributed less in the High group. The shares of ko00564 and ko00900 carried by *Enteractinococcus* decreased from 0.20 to 0.05 and from 0.20 to 0.07, respectively; that of ko00561 carried by *Mammaliicoccus* decreased from 0.15 to 0.07; and that of ko00072 carried by *Aerococcus* decreased from 0.24 to 0.03 (High/Low ratios, 0.13–0.43).

Carrier shifts did not simply track total pathway abundance. The High/Low ratios of total pathway TPM for the five pathways were 0.66, 0.38, 0.43, 0.23, and 0.56; thus, although the total abundance of all five pathways was lower in the High group, their main carriers shifted from Low- to High-quality-associated genera. Taxonomic abundance, total pathway abundance, and carrier composition therefore represent related but non-interchangeable levels of description, and the carrier level provides information that is not visible in total pathway abundance.

Because only six samples were available, we compared the observed grouping with all ten possible 3-versus-3 partitions of the samples (Table S5). For the non-muscone background metabolome, the observed grouping ranked first among the ten partitions in both between-group separation (PERMANOVA pseudo-F, 1.56 vs. ≤1.18 for the other partitions) and the number of features whose values completely separated the two groups (24,801 vs. ≤15,317). At the genus level, it ranked third in Aitchison-space separation and second in the number of completely separated genera. In contrast, the mean carrier-profile distance of the five core pathways ranked seventh, and comparable or larger distances were obtained for arbitrary partitions. Carrier composition therefore varies substantially among individuals, and the between-group carrier differences described above should be interpreted as directional shifts within this individual variation rather than as a quality-specific signal in terms of their magnitude.

### 3.5. Quality-Associated Metabolic Remodeling Extends from Muscone to the Lipid, Sterol, and Volatile Background

After muscone was removed from the 116,658 detected features, 116,657 background features remained for the integrated analysis. Initial screening with *p* < 0.05 and VIP > 1 resulted in 5132 features, of which 2367 were higher and 2765 were lower in the High group. Positive-ion LC–MS, negative-ion LC–MS, and GC–MS contributed 2693, 2411, and 23 features, respectively. The candidate set was dominated by low-confidence annotations: 4844 were Level 4, 155 were Level 2, 79 were Level 1, 19 were Level 3.1, and eight were Level 3.2; 27 lacked an annotation level, and 283 had non-empty compound names. After FDR correction, three co-eluting lipid-like signals with Q < 0.05 remained (all detected in negative-ion mode with annotation level 4), indicating that large-scale metabolic differences were mainly manifested as a directionally consistent chemical background rather than as concentrated changes in a limited number of high-confidence individual compounds.

**Figure 4:**
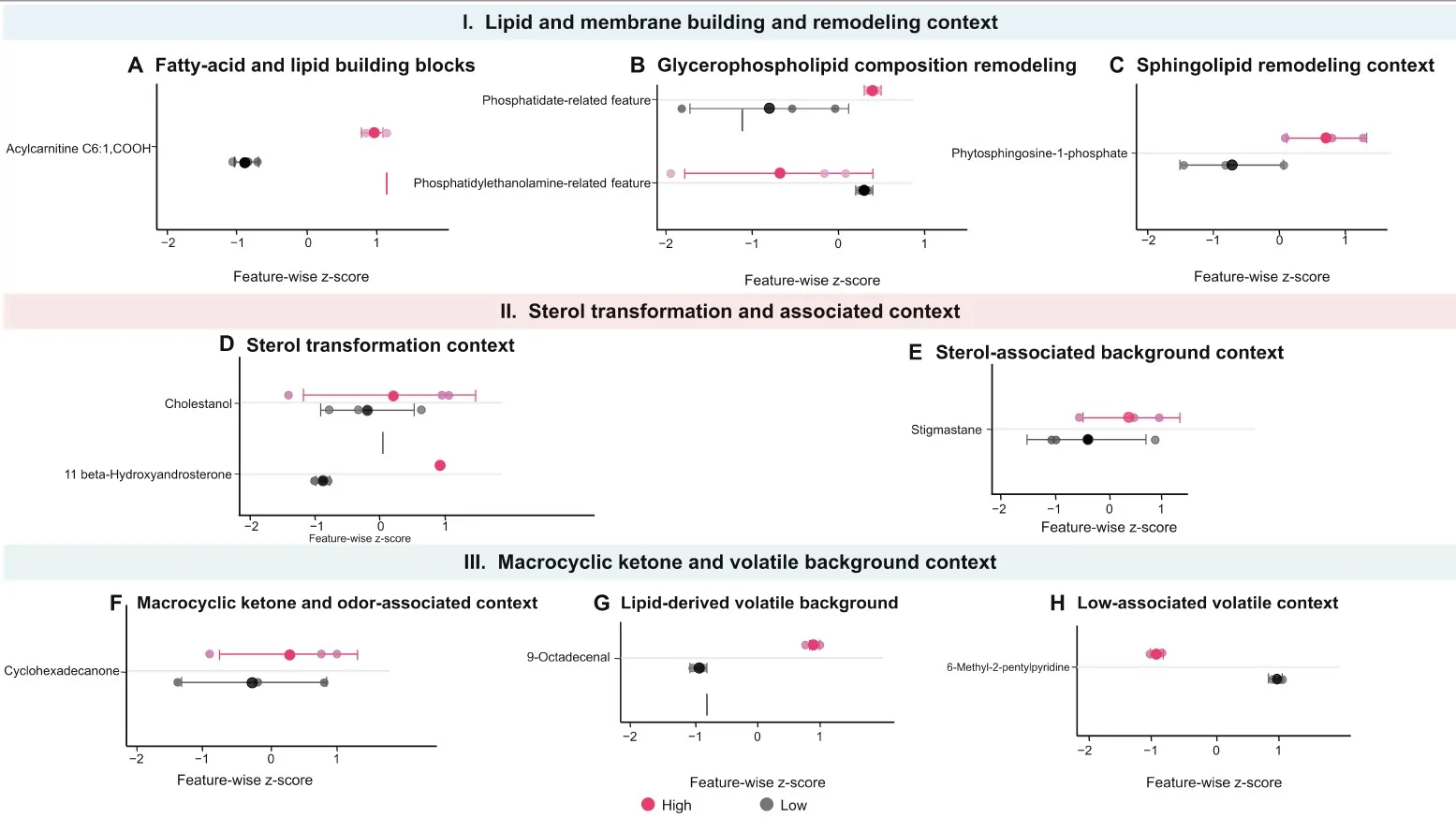
Coordinated remodeling of non-muscone background metabolic features along the quality axis: (**A**–**C**) fatty acid/lipid, glycerophospholipid, and sphingolipid modules; (**D**,**E**) sterol and related metabolic backgrounds; (**F**–**H**) macrocyclic ketones, lipid-derived volatiles, and Low-quality-associated volatiles. Pink and gray represent the High and Low groups, respectively; small points represent individual samples, whereas large points and connecting lines represent within-group summaries. The horizontal axis shows within-feature Z-scores, and compound names retain the candidate annotation levels in the source data.

Candidate features selected for the quality phenotype further converged into three interconnected chemical modules (Figure 4). In the lipid and membrane-component modules, the High−Low Z differences for acylcarnitine C6:1,COOH, a phosphatidic acid-related feature, and phytosphingosine 1-phosphate were +1.81, +1.37, and +1.44, respectively, with an LOO stability of 6/6 for all three. By contrast, a phosphatidylethanolamine-related feature shifted toward the Low group (Δ = −1.10, LOO 6/6). These opposing changes jointly link fatty acid activation, glycerophospholipid composition, and the sphingolipid background to quality-associated membrane-lipid remodeling.

The sterol and volatile modules extended along the same quality axis. 11β-Hydroxyandrosterone and stigmastane increased in the High group, with High−Low Z differences of +1.82 and +0.83, respectively, and both directions were retained in all six LOO analyses. Cholestanol increased by only 0.37, with an LOO stability of 4/6, indicating that individual differences remained within the sterol background. Among macrocyclic ketones and volatiles, cyclohexadecanone (Δ = +0.53, LOO 5/6) and 9-octadecenal (Δ = +1.82, LOO 6/6) were consistent with the High group, whereas 6-methyl-2-pentylpyridine was stably enriched in the Low group (Δ = −1.82, LOO 6/6). These changes distinguished high- and low-quality-associated samples by lipid–sterol–volatile combinations beyond muscone.

The Z-scores of the candidate features indicated their relative positions among the six samples, whereas the compound names retained their annotation levels in the source data. Although each candidate still requires confirmation with an authentic standard, concordant clustering across multiple chemical classes indicates that quality differentiation in natural musk involves remodeling of the entire secretory matrix rather than an isolated change in muscone content.

### 3.6. Multi-Omics Evidence Converges into Two Quality-Associated Ecological–Metabolic Configurations

Cross-layer integration showed that direct phenotypes, microbial consortia, functional carrier structure, and background metabolism were not isolated from one another; however, they were coordinately organized along the same quality axis. High-quality-associated samples jointly exhibited higher muscone content, lower moisture content, a consortium structure with *Corynebacterium* and *Actinobaculum* as key nodes, highly associated carrier configurations in the lipid, ketone body, and terpenoid pathways, and a metabolic background characterized by membrane lipids, sterols, and certain volatiles. Low-quality-associated samples were characterized jointly by reduced muscone content, increased moisture levels, replacement members, including *Enteractinococcus*, *Mammaliicoccus*, and *Aerococcus*, their functional carrier configurations, and different volatile backgrounds.

**Figure 5:**
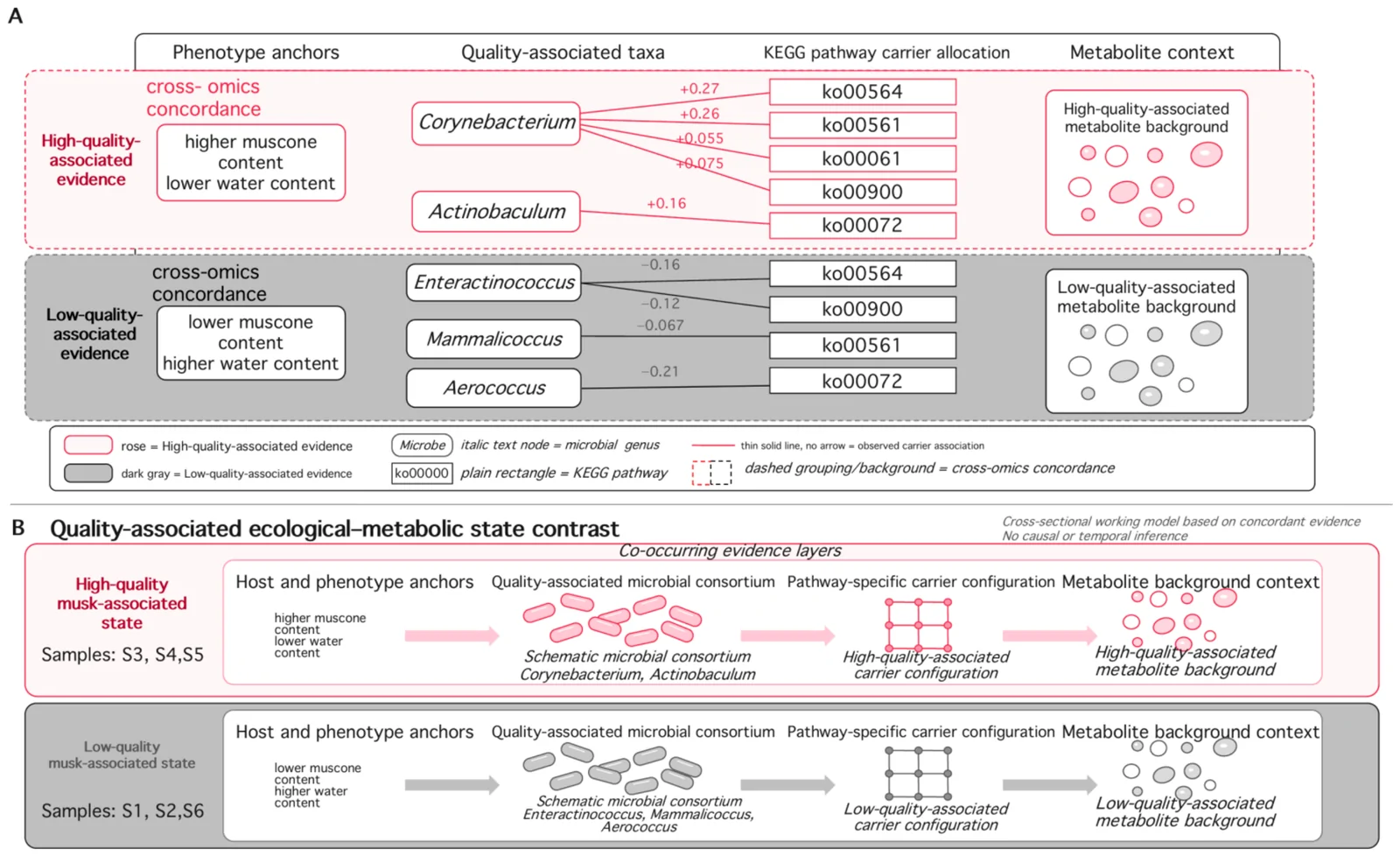
Multi-omics evidence integration and quality-associated ecological–metabolic configurations: (**A**) A continuous evidence chain composed of direct phenotypes, consortium members, pathway carrier shares, and background metabolic classes; values are the High−Low contribution shares of genera to the corresponding pathways. (**B**) Integrated comparison of the High- and Low-quality-associated configurations, showing the coordinated organization of phenotypes, microbial consortia, pathway-specific carrier configurations, and metabolic backgrounds. Lines in the figure indicate correspondence among levels of cross-sectional evidence.

As shown in Figure 5A, all five High-quality-associated carrier–pathway pairs (*Corynebacterium* with ko00564, ko00561, ko00061, and ko00900, and *Actinobaculum* with ko00072) retained their High−Low direction in all six LOO analyses (stability tier A), whereas the four Low-quality-associated pairs (*Enteractinococcus* with ko00564 and ko00900, *Mammaliicoccus* with ko00561, and *Aerococcus* with ko00072) retained it in five of six analyses and depended more on individual samples (tier B). In Figure 5B, the High configuration links these carriers to seven background features (a phosphatidate-related feature, phytosphingosine 1-phosphate, cholestanol, 11β-hydroxyandrosterone, stigmastane, 9-octadecenal, and cyclohexadecanone), whereas the Low configuration links its carriers to a phosphatidylethanolamine-related feature and 6-methyl-2-pentylpyridine, together with the dark, wet, and partly off-odor phenotype.

Accordingly, Figure 5A integrates traceable phenotypes, taxonomic members, pathway carrier shares, and metabolic classes into a continuous evidence chain, whereas Figure 5B summarizes two parallel quality-associated ecological–metabolic configurations.

## 4. Discussion

### 4.1. Direct Phenotypes Reveal the Matrix Basis of Natural Musk Quality Differentiation

The on-site sensory grading was confirmed by two directly measured indices that changed in opposite directions, with higher muscone and lower moisture in the High group. Coordinated changes in color, odor, and chemical composition have been reported among different types and qualities of musk [3,5], and the complex lipid- and sterol-rich matrix of natural musk was also supported by early chemical studies [2]. The dark color, moist texture, and off-odors of our low-quality samples resemble the abnormal black musk and the unqualified, cream-like musk with sour or unpleasant odors described in other captive populations [3,5], suggesting that the present grouping captures a recurrent quality contrast rather than a peculiarity of one farm. The present study further established opposing changes in muscone and moisture as the starting point for multi-omics interpretation, thereby grounding quality stratification in directly measurable chemical phenotypes.

Moisture is not a passive dilution variable in this system. Additionally, it affects secretion viscosity, oxygen diffusion, substrate accessibility, and release of volatile components, thereby linking the host-secreted matrix, local ecological niche, and sensory presentation. Because muscone is reported on a wet-mass basis, part of the muscone difference could reflect dilution by water. However, the two groups remained completely separated after conversion to a dry-mass basis (1.91–2.40% vs. 3.06–4.23%; Table S4), and dilution cannot explain the simultaneous changes in color, odor, community structure, and multiple classes of background metabolites. A more reasonable interpretation is that decreasing moisture and the accumulation of the principal aroma compound jointly indicated a transition in the overall state of the secretory matrix.

The high moisture content, dark color, and occasional undesirable odors of the low-quality associated samples further substantiate this matrix perspective. Food-spoilage ecology studies have shown that off-odors are typically collectively influenced by substrate composition, redox environment, and metabolism by complex microbial communities, rather than being a direct readout of a single dominant bacterium [38]. Consistent with the assessors’ descriptions, 6-methyl-2-pentylpyridine and propionic acid were higher in all three low-quality samples than in all three high-quality samples, and the putrefaction-related candidates N-acetylcadaverine, 5-aminopentanoic acid, and isovaleric acid were also directionally higher in the Low group; however, other putrefaction-associated candidates, such as skatole and butanoic acid, showed the opposite direction (Table S6). Because these annotations are unconfirmed, the chemical basis of the off-odor remains to be established with authentic standards. Therefore, in natural musk, sensory differences are both part of the quality phenotype and important clues for tracing changes in membrane lipids, sterols, and the volatile background.

### 4.2. Microbial Consortia Are the Basic Units of Quality-Associated Community Organization

Previous studies on forest musk deer have linked the mating status, intraglandular maturation stage, secretion period, and musk quality to changes in community composition [1,5,6,7]. Based on these findings, the present study demonstrated that quality-associated community differences in natural musk mainly manifest as consortium reorganization rather than as an overall increase or decrease in taxonomic diversity. Genus-level Shannon indices varied widely within groups, and the Aitchison space retained notable individual differences. However, the nine focal genera still formed two opposing sets of members along the quality axis. Therefore, taxonomic heterogeneity and directional consistency coexist at the consortium level.

This structure was particularly evident in the sample-level data. *Corynebacterium* was the strongest mean-positive member in the High group, but was not dominant in S3. The Low-group genera *Enteractinococcus* and *Proteus* were primarily present in S1 and S6, respectively. Specifically, different individuals can form similar quality-associated community configurations through different combinations of dominant members. Therefore, a microbial consortium more effectively explains the natural musk community than a single “core bacterium”: its stability derives from functional complementarity among members rather than from an identical taxonomic composition shared by all samples.

These results partly agree with, and partly extend, previous studies of musk microbiota (Table S7). The focal genera identified here belong to the three phyla (Actinobacteria, Firmicutes, and Proteobacteria) that dominated earlier 16S rRNA gene surveys [5,6,7]. *Corynebacterium* was overrepresented in the musk of unmated males, which contained more muscone [1], and dominated both early and mature musk [6], which is consistent with its High-quality-associated pattern here. *Proteus*, which characterized the low-quality sample S6, dominated the intermediate, semi-solid stage of musk maturation [6], suggesting that some Low-quality-associated members may be typical of incompletely matured musk. In contrast, Wang et al. [5] found *Corynebacterium* to be more abundant in unqualified, cream-like musk than in qualified musk, which is opposite to the direction observed here. Differences in quality definitions, sampling season, sequencing approach (16S rRNA gene amplicons vs. shotgun metagenomics), farm environment, and strain-level diversity within *Corynebacterium* may all contribute to such discrepancies. This inconsistency supports the view that no single genus is likely to serve as a universal quality marker and that the role of a genus depends on the community and matrix context in which it occurs.

This combinatorial organization is highly consistent with that of other animal scent-gland systems. Hyena social odors covary with glandular microbiota; symbiotic bacteria in giant pandas can participate in volatile-signal synthesis, and metabolome–microbiome integration at scent-marking sites also shows correspondence between multiple classes of volatiles and community structure [10,12,13]; in wild meerkats (*Suricata suricatta*), anal-gland volatiles likewise covary with bacterial assemblages [39]. Together, this cross-species evidence is consistent with the interpretation that animals and microorganisms in their secretions are interacting ecological units [40]. For forest musk deer, the present study advances this perspective by further examining whether quality-associated functions are retained when consortium members are replaced and to which members these functions are transferred.

### 4.3. Functional Carrier Redistribution Reveals Ecological Reorganization Beneath Total Pathway Abundance

A central observation of this study was that the same KEGG functional potential was carried by different genera in different samples and groups. The theory of functional redundancy highlights that community taxonomic and functional compositions do not individually correspond. Multiple taxa can encode the same function; however, their resource use strategies, spatial locations, and symbiotic relationships are not equivalent [14]. Functional redundancy is thought to stabilize community function under disturbance [41,42] and, in the human microbiome, arises from a genomic content network in which many taxa share the same genes [43]. Frameworks that trace community-level functional shifts back to specific taxa have further shown that the taxa driving a shift can differ markedly between functions [17]. A model study of the rumen further demonstrated that assigning metabolic functions to specific microbial members could extract mechanisms linked to host phenotypes from community differences [15]. Our data indicate that such carrier variation is also pronounced in natural musk.

Within this variation, the High and Low groups differed directionally in their main carriers: *Corynebacterium* and *Actinobaculum* carried larger shares of the lipid, ketone-body, and terpenoid pathways in the High group, whereas *Enteractinococcus*, *Mammaliicoccus*, and *Aerococcus* carried larger shares in the Low group (Section 3.4). However, the partition analysis showed that carrier-profile distances of similar magnitude also arise between arbitrary partitions of the six samples (Table S5). These paired shifts should therefore be regarded as candidate quality-associated carriers embedded in large individual variation rather than as a quality-specific carrier signature.

In addition, carrier changes occurred even when the total pathway TPM was lower in the High group. For example, the main carriers of all five core pathways shifted toward High-quality-associated genera, although the total abundance of these pathways was lower in the High group. Therefore, three distinct ecological questions are whether a pathway is present, how abundant it is, and who carries it. Two communities with similar total pathway abundance may still differ in the physiology, growth conditions, and spatial distribution of the organisms that encode a pathway, and thus in when and how strongly the pathway is used. Carrier analysis therefore adds a level of information that is not captured by conventional pathway abundance or enrichment analyses and provides an additional layer for linking taxonomic replacement to the metabolic background.

This carrier structure indicates that even when the overall functional potential changes slightly, changes in the cell types performing the functions and their co-occurring partners may reorganize substrate competition, metabolite exchange, and the local reaction environment, because taxa sharing a function generally differ in resource use, growth strategy, and interactions with co-occurring organisms [14,17,41]. It should be emphasized that the carrier matrix describes metagenomically encoded functional potential obtained directly from shotgun sequences rather than inferred from marker genes; it does not show whether the genes are expressed or whether the corresponding metabolic fluxes occur. Future studies should use strain genomes, metatranscriptomics, proteomics, and stable-isotope tracing to extend carrier shares to actual expression and metabolic flux.

### 4.4. Background Metabolic Remodeling Links Community Functions to the Aroma Matrix

After excluding muscone, quality differences were still mapped stably onto three chemical modules, lipids/membranes, sterols, and volatiles, indicating extensive matrix remodeling between the two groups. Natural musk has high levels of lipids and sterols [1,2]. Steroidogenesis-related factors in the musk gland and changes in blood metabolism during the secretion period further indicate that the host lipid–steroid supply is an important component of glandular secretion [44,45]. The phospholipid, sphingolipid, acylcarnitine, and steroid candidate features observed in this study jointly indicated a quality-associated secretory matrix state.

In high-quality-associated samples, acylcarnitine C6:1,COOH, phosphatidic acid, phytosphingosine 1-phosphate, 11β-hydroxyandrosterone, and 9-octadecenal were enriched in the same direction. Low-quality-associated samples showed increased phosphatidylethanolamine-related features and 6-methyl-2-pentylpyridine content. Phosphatidylethanolamine-related features may link membrane lipid turnover to lipid-derived volatiles, because the oxidation of unsaturated membrane fatty acids yields volatile aldehydes [46]. 6-Methyl-2-pentylpyridine may provide a testable chemical clue for the off-odors of some samples: alkylpyridines such as 2-pentylpyridine can form when lipid-oxidation aldehydes such as 2,4-decadienal react with amino acids [47], and ammonia-like notes were among the off-odors recorded by the assessors. Although candidate identities require confirmation with authentic standards, concordant changes across chemical classes provide stronger evidence of background metabolic remodeling than any individual metabolite.

Complex fermentation systems provide an informative reference for understanding this “consortium–function–flavor” relationship. Member interactions in cheese-rind communities can be reconstructed in model systems [48]. Kefir fermentation depends on microbial succession, metabolic cooperation, and spatiotemporal niche partitioning [49,50], and multi-omics studies of cheddar and surface-ripened cheeses show that changes in community structure occur synchronously with flavor metabolic profiles [51,52]. Substrate exchange, mutualism, and competition in mixed cultures jointly determine the final metabolic output [53,54]. Natural musk has a unique context of continuous host supply and the microenvironment of the gland sac; however, its quality may depend on the cooperative use of complex lipids and volatile precursors by the microbial consortia.

Comparisons with other musk-producing mammals support this interpretation. In a multi-omics comparison of forest musk deer and the muskrat (*Ondatra zibethicus*), whose musk glands evolved independently, the musk microbiota of both species was dominated by *Corynebacterium* and enriched in lipid-metabolizing functions, including enzymes of fatty acid degradation and ketone body metabolism [55]. Muskrat musk likewise contains fatty acids, esters, cholesterol, and macrocyclic ketones [56], and androgen-dependent musk synthesis in the muskrat scent gland involves fatty acid metabolism and terpenoid backbone biosynthesis [57]. The association of *Corynebacterium* with lipid, ketone-body, and terpenoid carriers in our High group is therefore consistent with a lipid-processing role of this genus that may be shared across musk-producing mammals, although its contribution in forest musk deer appears to depend on individual and matrix context.

### 4.5. Quality-Associated Ecological–Metabolic Configurations and Their Research Significance

Collectively, the evidence summarizes natural musk quality in two concurrent ecological–metabolic configurations. The high-quality-associated configuration comprised a “high-muscone–low-moisture” phenotype, a High-quality-associated microbial consortium, specific carrier structures in lipid/ketone-body/terpenoid pathways, and a coordinated lipid–sterol–volatile background. The low-quality-associated configuration was characterized by high moisture, low muscone, an alternative microbial consortium, and corresponding functional and volatile backgrounds. This framework incorporated a host-secreted matrix, community member identities, functional sources, and chemical output within a single system, providing a more complete biological explanation than a single-index quality evaluation.

The conceptual value of this model is in advancing natural musk quality research from asking “which bacteria or metabolites increase or decrease” to asking “which consortia, with what functional organization, correspond to which chemical configurations.” Functional carrier redistribution forms an intermediate layer between taxonomic composition and metabolic background, which may help explain why different samples can have different dominant bacteria but still exhibit consistent functional and metabolic characteristics along the quality axis. The resulting candidate carrier pairs and metabolic endpoints provide priority targets for developing quality-monitoring panels, isolation and cultivation systems, and mechanistic experiments. In practice, these results suggest three applications that require validation in larger cohorts. First, musk quality assessment could combine muscone with moisture and with candidate microbial and chemical indicators, such as the relative abundance of *Corynebacterium* versus *Enteractinococcus* and the off-odor-related feature 6-methyl-2-pentylpyridine. Second, monitoring the musk-gland microbiota and hygiene during live harvesting could provide an early warning of low-quality musk in captive breeding. Third, the candidate carrier genera provide targets for isolating strains that may be used to study, and eventually support, musk maturation.

Future studies should move from cross-sectional associations to process validation, with priorities derived directly from the present results: (i) repeated sampling of the same individuals across the musk secretion season, with sample sizes planned from the effect sizes reported here, to establish the temporal order of changes in phenotype, consortia, and carriers; (ii) quantification of muscone and moisture on both wet- and dry-mass bases, together with absolute microbial load; (iii) targeted quantification with authentic standards of the key candidate features, including 6-methyl-2-pentylpyridine, 11β-hydroxyandrosterone, 9-octadecenal, and cyclohexadecanone; (iv) metatranscriptomic and metaproteomic profiling of ko00564, ko00561, ko00072, and ko00900, combined with genome-resolved metagenomics, to test whether the candidate carriers express these pathways; and (v) isolation of *Corynebacterium*, *Actinobaculum*, *Enteractinococcus*, and *Proteus* strains and their culture, alone and as synthetic consortia, in a sterilized musk matrix to test their effects on lipids, sterols, and volatiles.

### 4.6. Study Limitations and Validation Pathways

This study has several limitations. First, it included six deer (three per group) from a single farm sampled at a single time point, so the findings are exploratory; nevertheless, previous musk microbiota studies have also analyzed 10–12 musk samples [1,5,6] (Table S7). Because mating status, season, and gland developmental stage influence musk microbiota and chemistry [1,6,7], the present findings apply to fully matured musk collected on one farm in February and should not be generalized to natural musk quality as a whole; sampling across farms and years, including other captive breeding farms such as those studied previously [1,4,5,6], is needed to test their generality. Second, the cross-sectional design cannot establish the temporal order or causality of ecological–metabolic changes. Third, the microbial data are relative abundances, and the KEGG carrier matrix reflects encoded potential rather than expression or activity; moreover, carrier-profile distances of similar magnitude occurred between arbitrary partitions of the samples (Table S5). Fourth, most metabolite names are candidate annotations that have not been confirmed with authentic standards. In addition, the sensory traits were recorded as categorical descriptors rather than measured instrumentally; future studies should add instrumental color measurement and gas chromatography–olfactometry. Fifth, muscone and moisture were reported on a wet-mass basis. The groups also remained separated for muscone on a dry-mass basis (Table S4); future studies should report muscone on both bases.

## 5. Conclusions

In this exploratory study of six forest musk deer from one farm, sampled in February, high-quality musk combined higher muscone, lower moisture, a distinct microbial consortium, and a coordinated lipid, sterol, and volatile background; this non-muscone background separated the two quality groups more strongly than any other partition of the six samples.

The same KEGG functional potential was carried by different genera in different individuals, and the main carriers of lipid, ketone-body, and terpenoid pathways shifted directionally between quality groups, although carrier composition also varied widely among individuals. Identifying which organisms carry a function therefore adds information beyond total pathway abundance. In practice, combining muscone and moisture with candidate microbial and odor-related markers may improve musk quality assessment and the management of captive musk deer; these candidates now require validation in larger, multi-farm, and longitudinal cohorts.

## Data Availability

Raw metagenomic sequences have been deposited in the Genome Sequence Archive (GSA) of the National Genomics Data Center, China National Center for Bioinformation (CNCB-NGDC), under project accession number CNP0009979, and raw GC–MS and LC–MS data and peak tables were deposited in MetaboLights under accession number MTBLS15284. Until the public accession numbers become available, the data may be obtained from the corresponding author upon reasonable request and with permission from the animal-sample provider.

## Supplementary Materials

The following supporting information can be downloaded at https://www.mdpi.com/article/10.3390/ani16213540/s1. Complete sample metadata, metagenomic quality control, full taxonomic composition, KEGG functional potential, metabolite annotation levels, *p*/Q values for differential features, exploratory cross-omics associations, supplementary ordination, sensitivity, and association results are provided in the Supplementary Materials. Table S4: Moisture, wet- and dry-basis muscone values, and effect sizes; Table S5: Rank of the observed grouping among all ten unique 3-versus-3 partitions of the six samples; Table S6: Candidate putrefaction- and off-odor-related features in the non-muscone background metabolome; Table S7: Comparison with previous studies of forest musk deer musk microbiota and chemistry.

## Abbreviations

The following abbreviations are used in this manuscript:

FDR	False discovery rate
GC–MS	Gas chromatography–mass spectrometry
KEGG	Kyoto Encyclopedia of Genes and Genomes
KO	KEGG Orthology
LC–MS	Liquid chromatography–mass spectrometry
LOO	Leave-one-out
PC	Principal component
PCA	Principal component analysis
PCR	Polymerase chain reaction
TPM	Transcripts/mapped reads per million normalized abundance
qPCR	Quantitative polymerase chain reaction
VIP	Variable importance in projection
